# Immune Mechanisms Involved in *Schistosoma mansoni*-Cathepsin B Vaccine Induced Protection in Mice

**DOI:** 10.3389/fimmu.2018.01710

**Published:** 2018-07-25

**Authors:** Alessandra Ricciardi, Nicholas H. Zelt, Kittipos Visitsunthorn, John P. Dalton, Momar Ndao

**Affiliations:** ^1^Department of Microbiology and Immunology, McGill University, Montreal, QC, Canada; ^2^Research Institute of the McGill University Health Center, Infectious Diseases and Immunity in Global Health (IDIGH) Program, National Reference Center for Parasitology, Montreal, QC, Canada; ^3^School of Biological Sciences, Medical Biology Centre (MBC), Queen’s University Belfast, Belfast, Ireland

**Keywords:** schistosomiasis, vaccine, adjuvant, antibodies, cellular immune response, cathepsin B

## Abstract

A vaccine against schistosomiasis would contribute to a long-lasting decrease in disease spectrum and transmission. Our previous protection studies in mice using *Schistosoma mansoni* Cathepsin B (Sm-Cathepsin B) resulted in 59 and 60% worm burden reduction with CpG oligodeoxynucleotides and Montanide ISA720 VG as adjuvants, respectively. While both formulations resulted in significant protection in a mouse model of schistosomiasis, the elicited immune responses differed. Therefore, in this study, we aimed to decipher the mechanisms involved in Sm-Cathepsin B vaccine-mediated protection. We performed *in vitro* killing assays using schistosomula stage parasites as targets for lung-derived leukocytes and serum obtained from mice immunized with Sm-Cathepsin B adjuvanted with either Montanide ISA 720 VG or CpG and from non-vaccinated controls. Lung cells and immune sera from the Sm-Cathepsin B + Montanide group induced the highest killing (63%) suggesting the importance of antibodies in cell-mediated parasite killing. By contrast, incubation with lung cells from Sm-Cathepsin B + CpG immunized animals induced significant parasite killing (53%) independent of the addition of immune serum. Significant parasite killing was also observed in the animals immunized with Sm-Cathepsin B alone (41%). For the Sm-Cathepsin B + Montanide group, the high level killing effect was lost after the depletion of CD4^+^ T cells or natural killer (NK) cells from the lung cell preparation. For the Sm-Cathepsin B + CpG group, high parasite killing was lost after CD8^+^ T cell depletion, and a reduction to 39% was observed upon depletion of NK cells. Finally, the parasite killing in the Sm-Cathepsin B alone group was lost after the depletion of CD4^+^ T cells. Our results demonstrate how the different Sm-Cathepsin B formulations influence the immune mechanisms involved in parasite killing and protection against schistosomiasis.

## Introduction

Schistosomiasis is one of the most important human parasitic infections. The number of infected individuals surpasses 200 million, and this number may be an underestimate of the true infection burden (1). The majority of infected individuals suffer from long-term schistosomiasis-related conditions such as developmental complications, anemia, and chronic pain while severe morbidity is represented by the hepatosplenic form of the disease (1–3). Praziquantel is currently the only drug used to treat schistosomiasis, and it is the mainstay of mass drug administration (MDA) programs which benefited from substantial pharmaceutical donations (1). However, the MDA programs’ impact on disease transmission has often been questioned, and field studies in Kenya have shown no evidence of an overall reduction in schistosome transmission after several rounds of praziquantel treatment (4). Maintained disease transmission is particularly a problem for phenotypically susceptible individuals (5) who do not acquire resistance to reinfection after multiple rounds of treatment. Moreover, the use of praziquantel is a double-edged sword as increasing the proportion of schistosomes exposed to the drug increases selection pressure on drug resistance. The possibility of praziquantel resistant parasite emergence has been discussed for years (6–10) and represents a valid concern as schistosomiasis treatment relies on this one drug. A multifaceted approach to schistosomiasis control that would include drug treatment, snail control, water sanitation, hygiene education, and better disease mapping is suggested (1, 11). However, the development of an anti-schistosome vaccine would significantly improve schistosomiasis control efforts, especially if it would be incorporated within this multifaceted approach.

Our group has focused on *Schistosoma mansoni* Cathepsin B (Sm-Cathepsin B), the most abundant cysteine peptidase found in the parasite gut, as a potential vaccine candidate. It is needed for schistosome development (12) and it functions in parasite nutrition through the digestion of blood macromolecules (13–17). It has been demonstrated that immunizations with Sm-Cathepsin B alone can significantly decrease parasite burden in a mouse model of schistosomiasis (18). With the addition of an adjuvant, either CpG dinucleotides (19) or Montanide ISA 720 VG (SEPPIC Inc., Fairfield, NJ, USA) (20), protection levels were increased when examining all forms of parasitological burden including worm, hepatic egg, and intestinal egg numbers. CpG dinucleotides are toll-like receptor 9 agonists and they promote a T-helper cell type 1 (Th1) response and have shown promise in vaccine formulations against various parasitic infections (21–25). Differently, Montanide ISA 720 VG is a squalene-based adjuvant which forms water-in-oil droplets that allow for slow antigen release at the site of injection. Montanide adjuvants are acceptable for use in humans, and they have been used in over 50 clinical trials (26–28). Although both formulations, Sm-Cathepsin B in combination with CpG and with Montanide ISA 720 VG, elicited significant protection in a mouse model of schistosomiasis, the immune responses which they generated differed. Sm-Cathepsin B with CpG stimulated a Th1-biased response (19) whereas the Montanide ISA 720 VG adjuvanted formulation yielded a mixed Th1/Th2 response (20). This suggests that the soluble and/or cellular effector mechanisms involved in the vaccine-mediated protection differ between formulations.

In the present study, we sought to determine the antibody-dependant and cellular effectors involved in mediating the protection elicited by our different Sm-Cathepsin B vaccine formulations. We focused on the lung stage parasites, schistosomula, since studies on radiation-attenuated vaccines have shown that this stage is susceptible to immune-mediated protection mechanisms (29, 30). Therefore, lung cells obtained from mice vaccinated with Sm-Cathepsin B formulations were studied *in vitro* for their ability to kill schistosomula in the presence or absence of antibodies. We are the first to report mechanistic data behind the protection observed with the different formulations of Sm-Cathepsin B.

## Materials and Methods

### Expression and Purification of Sm-Cathepsin B

Expression and purification of the Sm-Cathepsin B recombinant protein were carried out as previously reported (19). Briefly, the PichiaPink™ expression system (Thermo Fisher Scientific, Waltham, MA, USA) was used and yeast cells were cultured in buffered complex glycerol medium followed by induction in buffered complex methanol medium. Purification of the recombinant protein was performed *via* Ni-NTA chromatography (Ni-NTA Superflow by QIAGEN, Venlo, Limburg, Netherlands) and the elution was analyzed by Coomassie blue staining of polyacrylamide gel and western blot (Figure S1 in Supplementary Material).

### Immunization Protocol

Female, 6- to 8-week-old C57BL/6 mice were purchased from Charles River Laboratories (Senneville, QC, Canada). Six groups containing 12–16 mice each were immunized intramuscularly in the caudal thigh muscles with different formulations. Group 1 (saline control): mice received 50 µl of phosphate buffered saline (PBS) (Wisent Bio Products, Saint-Jean-Baptiste, QC, Canada). Group 2 (antigen control): mice were immunized with 20 µg of recombinant Sm-Cathepsin B. Group 3 (CpG adjuvant control): mice received 40 µg of synthetic oligodeoxynucleotides containing unmethylated CpG dinucleotides (Catalog# HC4039, Cedarlane, Burlington, ON, Canada). Group 4 (antigen and CpG experimental): mice were immunized with 20 µg recombinant Sm-Cathepsin B and 40 µg CpG adjuvant. Group 5 (Montanide adjuvant control): mice received a 70% volume formulation of Montanide ISA 720 VG (SEPPIC Inc., Fairfield, NJ, USA). Group 6 (antigen and Montanide experimental): mice were immunized with 20 µg recombinant Sm-Cathepsin B in a 70% volume formulation of Montanide ISA 720 VG. All mice received an initial immunization of their respective formulation at week 0 and were boosted with the same formulation at weeks 3 and 6. The mice were sacrificed 3 weeks after the final boost (week 9). Whole lungs and blood samples were collected from all animals. The experiment was repeated four additional times. All animal procedures were performed in accordance with Institutional Animal Care and Use Guidelines, and the Animal Use Protocol 7625 was approved by the Animal Care and Use Committee at McGill University.

### Lung Cell Suspension Preparation

Whole lungs were collected from each mouse at week 9. The lungs were cut into very small pieces and transferred into tubes containing 10 ml of collagenase digestion solution: 10 ml PBS (Wisent Bio Products), 300 U/ml collagenase type II (Worthington Biochemical Corporation, Lakewood, NJ, USA), 150 μ1 of 10 mg/ml stock solution of DNase I (Sigma Aldrich, St. Louis, MS, USA). The lungs were digested at 37°C for 45 min under constant shaking. The lung digests were transferred onto strainers into new tubes and sterile syringe plungers were used to push any remaining lung pieces through the strainers. The strainers were washed twice with 5 ml of Hank’s balanced salt solution (Wisent Bio Products). All tubes containing the lung digests were centrifuged for 5 min at 300 × *g* and 4°C. The supernatants were decanted. At this point, lungs belonging to mice from the same group were pooled (three to five lungs from the same group were pooled). The pooled cell pellets were resuspended using PBS to a final volume of 10 ml. The tubes were centrifuged for 5 min at 300 × *g* and 4°C. The supernatants were decanted, the pellets resuspended in 10 ml PBS, and centrifuged once again for 5 min at 300 × *g* and 4°C. The supernatants were decanted and the pellets were resuspended in 10 ml MACS buffer: PBS (Wisent Bio Products), 2 mM EDTA (Sigma Aldrich), 0.5% fetal bovine serum (FBS) (Wisent Bio Products). Cell numbers were determined by trypan blue exclusion before proceeding to the cell isolation step.

Mouse CD45 MicroBeads (Miltenyi Biotec, Germany) were used, following the manufacturer’s instructions, for the positive selection of leukocytes (CD45^+^ cells) from the lung cell suspensions. Briefly, pelleted cells were resuspended in 90 µl MACS buffer followed by the addition of 10 µl CD45 MicroBeads and a 15 min incubation at 4°C in the dark. The cells were then washed with 1.5 ml MACS buffer and centrifuged for 10 min at 300 × *g* and 4°C. Pellets were resuspended in 500 µl MACS buffer and applied to MS columns placed in an OctoMACS™ separator (Miltenyi Biotec). The columns were washed three times with 500 µl MACS buffer. Afterward, the columns were removed from the separator and placed on clean collection tubes. Then, 1 ml of MACS buffer was added to each column and the magnetically labeled cell fraction (CD45^+^ population) was flushed out by firmly applying the plunger supplied with the column.

### Cell Population Depletions

Additional Miltenyi Biotec mouse MicroBead kits were used to deplete different cell populations (Miltenyi Biotec): CD4 (L3T4) MicroBeads, CD8a (Ly-2) MicroBeads, CD49b (DX5) MicroBeads, and biotin labeled F4/80 antibody in combination with anti-biotin MicroBeads. For the CD4, CD8a, and CD49b MicroBeads, the procedures were similar. Briefly, the cell suspension was centrifuged at 300 × *g* for 10 min and then the supernatant was removed completely. The pelleted cells were resuspended in 90 µl MACS buffer followed by the addition of 10 µl of the respective MicroBeads and 15 min incubation at 4°C in the dark. The cells were then washed with 1.5 ml MACS buffer and centrifuged for 10 min at 300 × *g* and 4°C. Pellets were resuspended in 500 µl MACS buffer and applied to MS columns placed in an OctoMACS™ separator.

Since F4/80 MicroBeads were not available, the F4/80^+^ cell population was depleted using an indirect method. Briefly, cells were pelleted and the supernatant was removed. The cells were resuspended in MACS buffer and anti-F4/80-biotin (the manufacturer’s recommended antibody dilution was 1:10 for up to 10^6^ cells/50 μl of buffer). The resuspended cells were incubated in the dark at 4°C for 10 min. The cells were washed with 1.5 ml MACS buffer and centrifuged for 10 min at 300 × *g* and 4°C. Pellets were resuspended in 500 µl MACS buffer and applied to MS columns placed in an OctoMACS™ separator. For all of the performed depletions, the columns were washed three times with 500 µl MACS buffer. Unlabeled cells passed through the column whereas the magnetically labeled CD4^+^, CD8a^+^, CD49b^+^, or F4/80^+^ cells were retained within the columns. Therefore, the total column effluent represented the depleted population. All of the cell depletions were followed by CD45 positive selection as previously explained.

### Flow Cytometry

Flow cytometry analysis was performed in order to confirm the specific cell population depletions. The cell suspension was centrifuged for 5 min at 400 × *g* and 4°C. The cells were washed with 200 µl PBS and centrifuged for 5 min at 400 × *g* and 4°C. The cells were stained with viability dye (eBioscience, San Diego, CA, USA) (0.1 μl/10^6^ cells in 100 µl PBS) and incubated for 30 min at 4°C in the dark. After the incubation, the cells were washed three times with 200 µl PBS + 1% FBS. The supernatant was removed, 50 µl of BD Mouse Fc block (2.4G2 Ab) (BD Biosciences, Mississauga, ON, Canada) (1 μl/10^6^ cells in 50 µl PBS) was added, and the cells were incubated in the dark for 10 min at 4°C. Next, 50 µl of the surface stain containing CD45-Buv395 Clone 30-F11 (BD Biosciences), CD4-FITC Clone RM4-5 (eBioscience), CD8a-PerCP-Cy5.5 Clone 53-6.7 (eBioscience), CD49b-PE Clone DX5 (eBioscience), F4/80-APC Clone BM8 (eBioscience) antibody mixture was added, and the cells were incubated in the dark at 4°C for 20 min. The manufacturers’ recommended antibody dilutions were used. The cells were centrifuged for 5 min at 400 × *g* and 4°C, and then washed with 200 µl PBS + 1% FBS. The supernatant was removed; the cells were resuspended in 100 µl IC Fix buffer (eBioscience) and incubated overnight at 4°C. The stained cells were acquired with a BD LSRFortesa (BD Biosciences). Flow cytometry analysis was performed using FlowJo (Tree Star, Inc., USA).

### Schistosomula Preparation

*Biomphalaria glabrata* snails infected with the *S. mansoni* Puerto Rican strain were obtained from the Schistosomiasis Resource Center of the Biomedical Research Institute (Rockville, MD, USA). Cercariae were collected by placing infected snails in a beaker containing water and shining a strong light above the beaker for 2 h. The collected cercariae were incubated on ice, in the dark for 1 h. The settled cercariae were resuspended in 8 ml of alpha-MEM (Thermo Fisher Scientific). The cercariae were vortexed for 1 min, rested on ice for 3 min, and finally vortexed again for 1 min. The detached parasite bodies could be observed under a microscope and collected using a Pasteur pipette. The parasites were then incubated on ice for 10 min in order to allow the parasite to pellet. The pellet was washed with OPTI media [Opti-MEM Reduced Serum Media (Thermo Fisher Scientific), 0.25 μg/ml fungizone (Thermo Fisher Scientific), 100 μg/ml streptomycin (Thermo Fisher Scientific), 100 U/ml penicillin (Thermo Fisher Scientific)], and then incubated on ice for 8 min. The pellet was washed a second time with OPTI media and incubate once again on ice for 8 min. A final wash using OPTI media with a reduced antibiotic concentration [Opti-MEM Reduced Serum Media (Thermo Fisher Scientific), 0.25 μg/ml fungizone (Thermo Fisher Scientific), 10 μg/ml streptomycin (Thermo Fisher Scientific), 10 U/ml penicillin (Thermo Fisher Scientific), 6% FBS (Wisent Bio Products)] was performed followed by an 8 min incubation on ice. The parasite pellet was resuspended in OPTI media with a reduced antibiotic concentration and schistosomula were plated in 96-well plates.

### *In Vitro* Killing Assay

For the *in vitro* killing assay, the isolated lung cells were the effector cells and the transformed schistosomula were the targets. In 96-well plates, approximately 60 schistosomula were added to each well. The different cell populations (CD45^+^, CD45^+^CD4^−^, CD45^+^CD8^−^, CD45^+^CD49b^−^, CD45^+^F4/80^−^) were seeded in 96-well plates as 1 × 10^5^ cells/well in a 100 µl cell suspension. The different incubation conditions included media, cells, serum, cells + pre-immune serum, and cells + immune serum. Pre-immune serum was collected by saphenous bleed prior to the first immunization at week 0, and immune serum was collected by cardiac puncture at week 9. Endpoint titers of Sm-Cathepsin B specific total IgG for the experimental groups, antigen + CpG and antigen + Montanide, were above 120,000 (19, 20). Fifty microliters of serum were added to designated wells to obtain a 1:4 dilution. Incubation at 56°C for 30 min was performed in order to heat inactivate the serum. Every test was plated in duplicate. The plates were incubated for 24 h at 37°C and 5% CO_2_, after which, the percentage of dead schistosomula was determined by microscopic examination of motility, granularity, shape integrity, and uptake of methylene blue dye by the schistosomula.

### Statistical Analysis

Data generated from this study were analyzed by one-way analysis of variance followed by Tukey multiple comparison post-test. Two-way analysis, followed by Bonferroni post-test, was used when comparing original and depleted samples. *p* Values less than 0.05 were considered significant.

## Results

### *In Vitro* Parasite Killing in the Presence of Lung Cells and Serum

**Figure 1:**
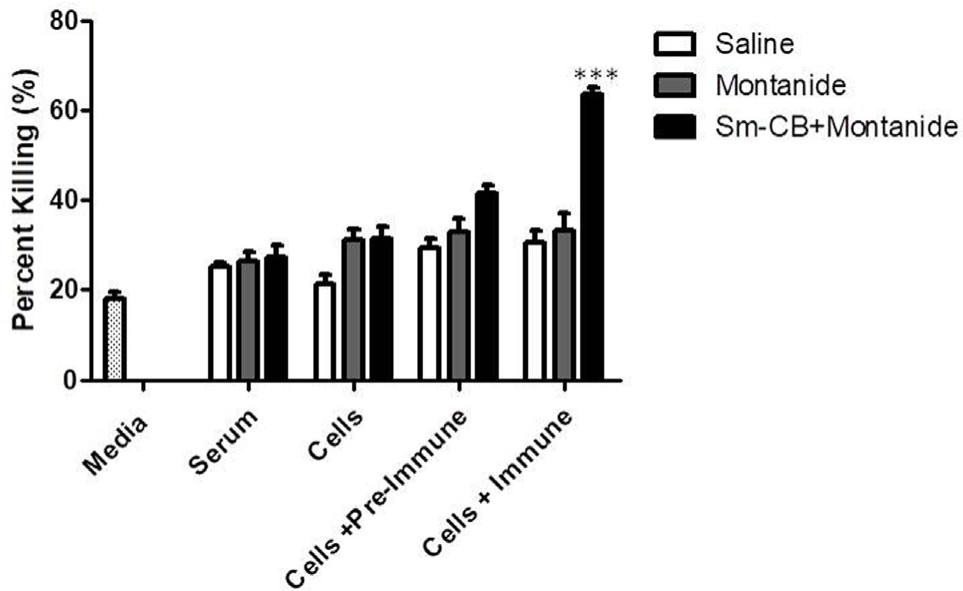
Schistosomula death with *Schistosoma mansoni* Cathepsin B (Sm-Cathepsin B) + Montanide. Sixty mechanically transformed schistosomula were incubated for 24 h at 37°C, 5% CO_2_ in the presence of media, serum, cells, cells + pre-immune serum, or cells + immune serum. CD45^+^ lung cells and serum were collected from the different immunized mouse groups: saline, Montanide ISA 720 VG, and Sm-Cathepsin B + Montanide ISA 720 VG. Percent parasite killing was determined for all groups and conditions. *n* = 14 for all groups. ***p ≤ 0.001.

**Figure 2:**
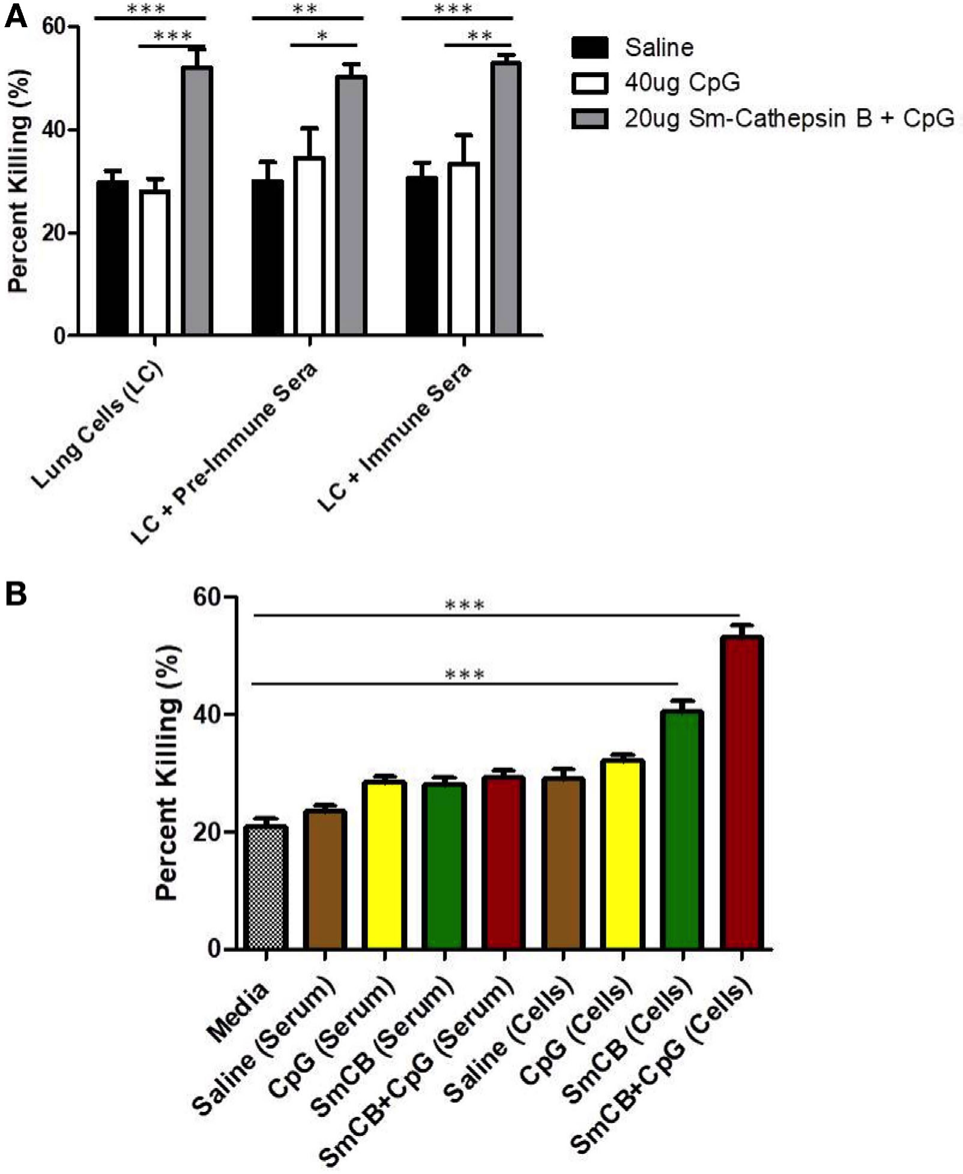
Schistosomula death with *Schistosoma mansoni* Cathepsin B (Sm-Cathepsin B) alone and Sm-Cathepsin B + CpG. **(A)** Sixty mechanically transformed schistosomula were incubated for 24 h at 37°C, 5% CO_2_ under different conditions which included media, serum, cells, cells + pre-immune serum, or cells + immune serum. CD45^+^ lung cells and serum were collected from the different immunized mouse groups: saline, CpG dinucleotides, Sm-Cathepsin B alone, and Sm-Cathepsin B + CpG. Percent parasite killing was determined for all groups and conditions. *n* = 5 for all groups. **(B)** Schistosomula incubation conditions focused on media, serum, and cells as the addition of immune serum to cells did not alter larvicidal effect. The checkered column represents the media control. The brown columns represent serum or cell conditions for the saline group. The yellow columns represent serum or cell conditions for the CpG adjuvant group. The green columns represent serum or cell conditions for the Sm-Cathepsin B group. The red columns represent the serum or cell conditions for the Sm-Cathepsin B + CpG group. *n* = 14 for all groups. ***p ≤ 0.001.

In order to investigate the effector mechanisms involved in vaccine-mediated protection, an *in vitro* assay was employed which involved culturing schistosomula in the presence of immune effector cells with/without serum from vaccinated mice. Schistosomula were incubated in media alone in order to establish background parasite death. We first showed that heat inactivated serum alone from the different animal groups had no significant effect on parasite viability in culture; thus, antibodies generated by vaccination alone are not cytotoxic to the schistosomula (Figures 1 and 2). The parasites were also incubated with lung cells alone, lung cells + pre-immune serum, and lung cells + immune serum obtained from the different control and experimental animal groups. In a separate set of experiments, lung lavage cells were used instead of cells isolated from whole lungs; however, this did not lead to novel observations worth pursuing (Figure S2 in Supplementary Material).

The experimental vaccine formulation of Sm-Cathepsin B and Montanide ISA 720 VG was compared to the saline control and the Montanide adjuvant control. As shown in Figure 1, the highest percentage of parasite killing (63%) was observed when schistosomula were incubated in the presence of both lung cells and immune serum from the Sm-Cathepsin B and Montanide ISA 720 VG group. The percentage of parasite killing obtained with this condition and group was significantly higher (*p* < 0.001) when compared to the control groups. Figure S3 in Supplementary Material depicts the reduced schistosomula viability in the wells containing lung cells and immune serum from the Sm-Cathepsin B + Montanide ISA 720 VG animals compared to the control wells. These data demonstrate that the addition of immune serum to cells from the experimental group is necessary to achieve significant levels of parasite killing. Therefore, an antibody-dependent cell-mediated cytotoxicity (ADCC) effect may explain the mechanism of protection in mice vaccinated with Sm-Cathepsin B and Montanide ISA 720 VG.

Next, immune serum and cells taken from mice vaccinated with Sm-Cathepsin B alone and the Sm-Cathepsin B + CpG formulation were compared to those taken from mice given saline or CpG adjuvant only. Both groups containing Sm-Cathepsin B (with CpG adjuvant and unadjuvanted) had increased parasite killing compared to the saline and adjuvant controls (Figure 2). This increased parasite toxicity was independent of the addition of immune serum to the lung cells (Figure 2A), indicating that the high larval killing observed with the Sm-Cathepsin B and Sm-Cathepsin B + CpG groups is dependent on cellular effectors. Therefore, subsequent observations focused on the presence of immune cells from the vaccinated animals. The percent parasite killing observed with cells from the Sm-Cathepsin B group, 41%, was significantly higher than all conditions with control groups (*p* < 0.001) (Figure 2B). The schistosomula killing recorded with the cells from the Sm-Cathepsin B + CpG group, 53%, was significantly higher compared to the killing obtained with the control groups as well as that obtained with the Sm-Cathepsin B alone group (*p* < 0.001) (Figure 2B). Examples of parasites with reduced viability in the presence of cells taken from the Sm-Cathepsin B + CpG group can be seen in Figure S4 in Supplementary Material. These observations suggest that the parasite killing effects elicited by immunizations with Sm-Cathepsin B and Sm-Cathepsin B + CpG are mediated by cellular effectors.

### Parasite Killing Post Cell Population Depletions

In order to begin dissecting the mechanisms involved in Sm-Cathepsin B vaccine-mediated protection, *in vitro* parasite killing assays were performed using lung cell preparations that had undergone depletions of specific immune cell populations. Once again the schistosomula were incubated in conditions that included lung cells alone, lung cells + pre-immune serum, and lung cells + immune serum. All of the cell population depletions were confirmed by flow cytometry (Figure S5 in Supplementary Material) (Table 1). The different cell population depletions in the saline control and adjuvant control groups had no effect on parasite viability.

**Table 1:** Depletion of cell populations in *Schistosoma mansoni* Cathepsin B (Sm-Cathepsin B) + Montanide, Sm-Cathepsin B + CpG, and Sm-Cathepsin B groups.

	Sm-Cathepsin B + Montanide		Sm-Cathepsin B + CpG		Sm-Cathepsin B	
	Original sample (%)	Depleted sample (%)	Original sample (%)	Depleted sample (%)	Original sample (%)	Depleted sample (%)
CD4^+^	12.70	1.48	19.50	0.89	16.80	0.94
CD8^+^	9.83	1.38	21.10	2.96	13.50	1.37
F4/80^+^	74.60	1.97	60.90	3.95	59.50	2.00
CD49^+^	4.65	0.58	5.72	0.31	6.18	0.37

**Figure 3:**
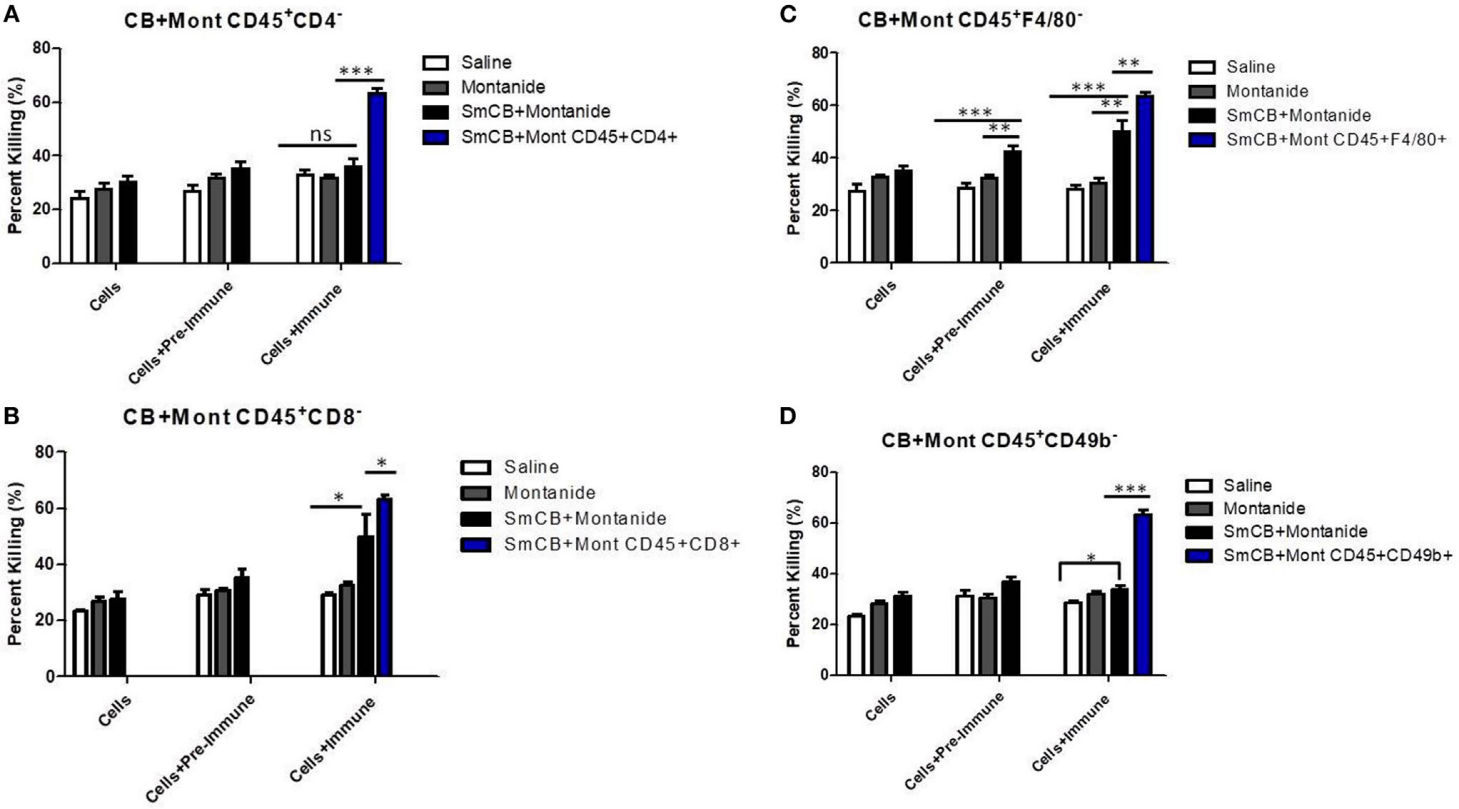
*Schistosoma mansoni* Cathepsin B (Sm-Cathepsin B) + Montanide: parasite killing after the depletion of different cell populations. Sixty mechanically transformed schistosomula were incubated 24 h at 37°C, 5% CO_2_ under different conditions which included cells, cells + pre-immune serum, and cells + immune serum. Lung cells and serum were collected from the different immunized mouse groups: saline, Montanide ISA 720 VG, and Sm-Cathepsin B + Montanide ISA 720 VG. The different immune cell depletions were as follows: **(A)** parasite killing was analyzed for the control groups and the experimental group after CD4 cell depletion; saline *n* = 10, Montanide *n* = 10, Sm-Cathepsin B + Montanide *n* = 14. Furthermore, parasite killing was compared between Sm-Cathepsin B + Montanide CD45^+^CD4^−^ and Sm-Cathepsin B + Montanide CD45^+^CD4^+^ (*n* = 14). **(B)** Parasite killing was analyzed for the control groups and the experimental group after CD8 cell depletion; *n* = 5 for all groups. Furthermore, parasite killing was compared between Sm-Cathepsin B + Montanide CD45^+^CD8^−^ and Sm-Cathepsin B + Montanide CD45^+^CD8^+^ (*n* = 14). **(C)** Parasite killing was analyzed for the control groups and the experimental group after F4/80 cell depletion; saline *n* = 7, Montanide *n* = 7, Sm-Cathepsin B + Montanide *n* = 11. Furthermore, parasite killing was compared between Sm-Cathepsin B + Montanide CD45^+^F4/80^−^ and Sm-Cathepsin B + Montanide CD45^+^F4/80^+^ (*n* = 14). **(D)** Parasite killing was analyzed for the control groups and the experimental group after CD49b cell depletion; saline *n* = 7, Montanide *n* = 7, Sm-Cathepsin B + Montanide *n* = 11. Furthermore, parasite killing was compared between Sm-Cathepsin B + Montanide CD45^+^CD49b^−^ and Sm-Cathepsin B + Montanide CD45^+^CD49b^+^ (*n* = 14). ns: *p* > 0.05, *p ≤ 0.05, **p ≤ 0.01, ***p ≤ 0.001. The white columns represent data from the saline group post depletion. The gray columns represent data from the Montanide adjuvant group post depletion. The black columns represent data from the Sm-Cathepsin B + Montanide group post depletion. The single blue columns represent the undepleted Sm-CathepsinB + Montanide sample for the immune serum + cells culture condition.

When CD4^+^ T cells were depleted, the parasite killing observed in the presence of serum and lung cells obtained from mice in the Sm-Cathepsin B + Montanide ISA 720 VG group was reduced from 63 to 36% which was similar to the levels of killing observed in the control groups. Moreover, the presence of immune serum had no effect on the percentage of schistosomula killing (Figure 3A). These observations indicate that CD4^+^ T cells are important effectors mediating the antibody-dependent parasite killing in Sm-Cathepsin B + Montanide ISA 720 VG immunized mice.

Depletion of CD8^+^ cells also significantly decreased the parasite killing in the experimental group, from 63 to 50% (*p* < 0.05) (Figure 3B). However, in this case, the presence of immune serum in the CD8^+^ depleted lung cells resulted in significantly higher killing compared to the absence of immune serum and to the saline control group (*p* < 0.05) (Figure 3B). This observation suggests that CD8^+^ T cells participate in parasite killing, although, they are not the primary immune cell effectors.

Depletion of F4/80^+^ cells (highly expressed on marophages in the lungs) resulted in 50% parasite viability (*p* < 0.01) (Figure 3C). The highest level of killing was observed when F4/80-depleted lung cells were incubated with immune serum (Figure 3C). The antibody-dependent effect suggested that F4/80^+^ cells are not the main mediators for the parasite killing mechanism elicited by Sm-Cathepsin B + Montanide ISA 720 VG immunizations.

Upon the depletion of natural killer (NK) cells (CD49b^+^), the percentage of parasite killing in the experimental group was reduced to levels comparable to the controls (34%) (Figure 3D). In this situation, the addition of immune serum to the NK-depleted lung cells did not increase parasite killing (Figure 3D). These observations indicate a role for NK cells in mediating parasite killing elicited by Sm-Cathepsin B + Montanide ISA 720 VG immunizations.

**Figure 4:**
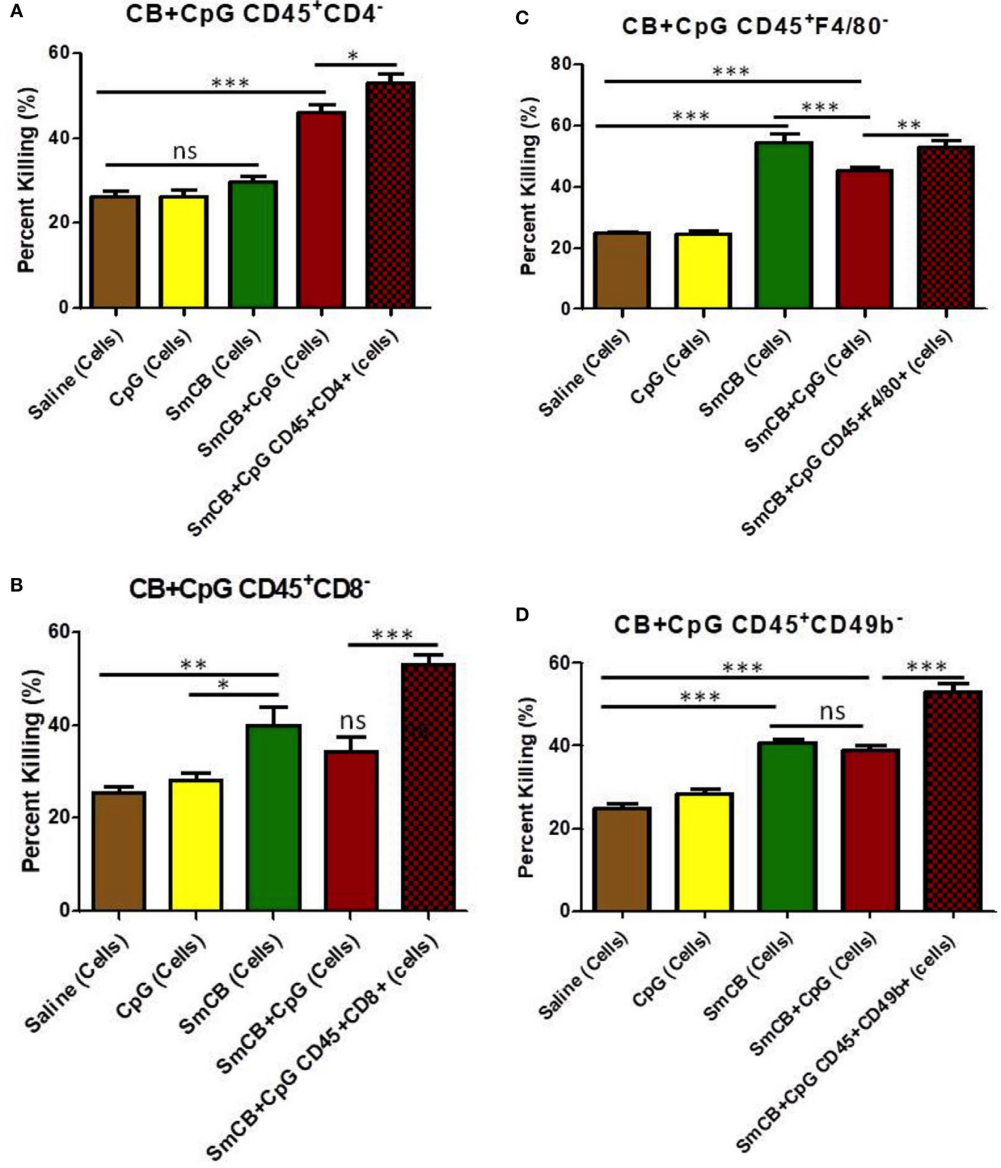
*Schistosoma mansoni* Cathepsin B (Sm-Cathepsin B) alone and Sm-Cathepsin B + CpG: parasite killing after the depletion of different cell populations. Sixty mechanically transformed schistosomula were incubated 24 h at 37°C, 5% CO_2_. Lung cells were collected from the different immunized mouse groups: saline, CpG dinucleotides, Sm-Cathepsin B, and Sm-Cathepsin B + CpG. The different depletions and comparisons were as follows: **(A)** parasite killing was analyzed for all groups after CD4 cell depletion; saline *n* = 10, CpG. *n* = 10, Sm-Cathepsin B *n* = 10, Sm-Cathepsin B + CpG *n* = 14. In addition, parasite killing was compared between Sm-Cathepsin B + CpG CD45^+^CD4^−^ and Sm-Cathepsin B + CpG CD45^+^CD4^+^ (*n* = 14). **(B)** Parasite killing was analyzed for all groups after CD8 cell depletion; *n* = 5 for all groups. In addition, parasite killing was compared between Sm-Cathepsin B + CpG CD45^+^CD8^−^ and Sm-Cathepsin B + CpG CD45^+^CD8^+^ (*n* = 14). **(C)** Parasite killing was analyzed for all groups after F4/80 cell depletion; saline *n* = 7, CpG *n* = 7, Sm-Cathepsin B *n* = 7, Sm-Cathepsin B + CpG *n* = 11. In addition, parasite killing was compared between Sm-Cathepsin B + CpG CD45^+^F4/80^−^ and Sm-Cathepsin B + CpG CD45^+^F4/80^+^ (*n* = 14). **(D)** Parasite killing was analyzed for all groups after CD49b cell depletion; saline *n* = 7, CpG *n* = 7, Sm-Cathepsin B *n* = 7, Sm-Cathepsin B + CpG *n* = 11. In addition, parasite killing was compared between Sm-Cathepsin B + CpG CD45^+^CD49b^−^ and Sm-Cathepsin B + CpG CD45^+^CD49b^+^ (*n* = 14). ns: not significant, *p* > 0.05, *p ≤ 0.05, **p ≤ 0.01, ***p ≤ 0.001. The brown columns represent data from the saline group post depletion. The yellow columns represent data from the CpG adjuvant group post depletion. The green columns represent data from the Sm-Cathepsin B group post depletion. The red columns represent data from the Sm-Cathepsin B + CpG group post depletion. The single red checkered columns represent the undepleted Sm-CathepsinB + CpG sample for the immune serum + cells culture condition.

Levels of parasite killing observed with cells obtained from the Sm-Cathepsin B + CpG mice were slightly reduced from 53 to 46% after CD4^+^ T cell depletion (Figure 4A). Larval killing in the Sm-Cathepsin B + CpG group was still significantly higher compared to the control groups (Figure 4A). The conservation of schistosomula killing upon CD4^+^ T cell depletion suggests that other cellular effectors are involved in the protection mediated by Sm-Cathepsin B + CpG immunizations. By contrast, the percentage of parasite death with lung cells from the unadjuvanted Sm-Cathepsin B immunized animals decreased to levels comparable to controls (30%) upon depletion of CD4^+^ T cells (*p* < 0.001) (Figure 4A). These data indicate that CD4^+^ T cells are necessary effectors in the protection mediated by unadjuvanted Sm-Cathepsin B immunizations.

Parasite killing was reduced to 34% in the Sm-Cathepsin B + CpG after CD8^+^ T cell depletion (*p* < 0.001) (Figure 4B). This level was comparable to that observed in the saline and CpG adjuvant control groups (Figure 4B) and indicates an important role for CD8^+^ T cells in mediating parasite killing in Sm-Cathepsin B + CpG immunized mice. By contrast, larval killing was maintained (40%) in the Sm-Cathepsin B alone group following CD8^+^ T cell depletion suggesting that, in this group, CD8^+^ T cells have a negligible effect on the protection (Figure 4B).

When F4/80^+^ cell populations were depleted from lung cells taken from the Sm-Cathepsin B and Sm-Cathepsin B + CpG groups, significantly higher parasite killing compared to the saline and adjuvant control groups was maintained (*p* < 0.001) (Figure 4C). Incubation of schistosomula with depleted F4/80^+^ cells from the CpG-adjuvanted experimental group resulted in parasite killing levels that were slightly reduced compared original observations; 53–45% (*p* < 0.01) (Figure 4C). This suggests that although these cells are not required to maintain high levels of larval death, they may contribute to the parasite killing mechanism elicited by Sm-Cathepsin B + CpG immunizations. By comparison, F4/80^+^ cell depletion from lung cells taken from the unadjuvanted Sm-Cathepsin B group increased parasite killing to 55% (*p* < 0.001) (Figure 4C). This depletion resulted in significantly higher levels of parasite death in the unadjuvanted group compared to the CpG-adjuvanted group (*p* < 0.001) (Figure 4C).

Depletion of NK cells resulted in schistosomula killing observed in the Sm-Cathepsin B + CpG group decreasing from 53 to 39% (*p* < 0.001) (Figure 4D). Although this level of killing was still significantly higher than that recorded for the saline and adjuvant control groups, the substantial decrease suggests that NK cells may belong to a network of cellular effectors mediating vaccine induced protective effects (Figure 4D). Furthermore, upon the depletion of NK cells, the parasite killing elicited by the Sm-Cathepsin B + CpG group was no longer significantly higher than the killing observed with the Sm-Cathepsin B alone group (Figure 4D). The percentage of parasite killing with the cells from the Sm-Cathepsin B alone animals was unaffected by the depletion of NK cells (41%) (Figure 4D). The maintenance of schistosomula killing upon NK cell depletion suggests that other cellular effectors are involved in protection mediated by unadjuvanted Sm-Cathepsin B immunizations.

## Discussion

Our past vaccine studies using the candidate Sm-Cathepsin B induced promising protection levels against schistosomiasis in mice whether formulated with Montanide ISA 720 VG or CpG dinucleotides adjuvants (19, 20). Although parasite burden reductions were similar between the different formulations tested, we found that the immune responses elicited were entirely different. Immunizations with recombinant Sm-Cathepsin B in combination with Montanide ISA 720 VG resulted in a mixed Th1/Th2 antigen-specific response (20) while immunizations with the antigen formulated in CpG yielded a biased Th1 response (19). However, both formulations elicited robust Sm-Cathepsin B specific total IgG antibody production pre-challenge. The protective role of antibodies in schistosomiasis has been demonstrated and antibody titers at the time of cercarial challenge are inversely correlated with worm burden (31). The importance of antibodies is further supported by the demonstration that protection in baboons immunized with radiation-attenuated cercariae is proportional to antibody titers (31). Moreover, several passive transfer studies have shown that both wild type and immunologically deficient animals have decreased parasite burden and pathology when they receive antibodies from chronically infected or immunized wild type animals (32–35). Therefore, the elevated antigen-specific IgG titers elicited by the previously tested formulations of recombinant Sm-Cathepsin B in the presence of CpG dinucleotides or Montanide (19, 20) (endpoint titers > 120,000) may be a crucial factor contributing to the decreased parasite burden observed at the time of perfusion and organ collection. In order to determine whether protection is associated with antibody-dependent effectors, ADCC was analyzed in this study.

The schistosomula are the most vulnerable to an immune attack as they migrate through the lungs, and the level of resistance has an important effect on the establishment of infection (36, 37). Therefore, we used schistosomula as targets in order to determine *in vitro* cytotoxicity effects of cells in the presence of antibodies. We found that the highest level of parasite killing was observed when CD45^+^ lung cells from the Sm-Cathepsin B + Montanide immunized animals were incubated in the presence of immune serum. The requirement of both lung cells and immune serum from the immunized mice for high parasite killing indicates that the mechanism involved is antibody dependent and cell mediated. Although high parasite killing was also observed with CD45^+^ lung cells from the Sm-Cathepsin B + CpG mice, this effect was not dependent on the presence of immune serum. All incubation conditions containing lung cells from the Sm-Cathepsin B + CpG vaccinated mice (cells alone, cells + pre-immune serum, and cells + immune serum) led to parasite killing levels that were significantly higher than those observed with cells from the control groups. These observations suggest that the mechanism involved in protection of these mice is cell dependent but antibody independent. This significant difference between the two Sm-Cathepsin B formulations is likely linked to the IgG subclasses involved. Although both formulations elicited robust antigen-specific IgG titers, IgG1 was the dominant subclass present in animals immunized with Sm-Cathepsin B + Montanide, whereas IgG2c was the dominant subclass in animals that received the CpG adjuvanted formulation (19, 20).

Antibody-dependent cell-mediated cytotoxicity is a known effector function of IgG1 antibodies as they are efficiently bound by Fc gamma receptors (FcγRs) on effector cells (38). On the other hand, IgG2c antibodies are not associated with ADCC properties. The antibody-independent cellular effect observed in the Sm-Cathepsin B + CpG immunized animals was not unexpected. CpG dinucleotides have been shown to enhance both innate and adaptive cellular responses (39). Activation of TLR9 by CpG dinucleotides stimulates the migration of plasmacytoid dendritic cells to T cell zones of lymphoid organs where they upregulate the expression of co-stimulatory molecules and promote strong Th1 CD4 T cell and cytotoxic T lymphocyte (CTL) responses (39).

Different cell populations were depleted in order to decipher the main effectors mediating protection in vaccinated animals. It is important to note that not all cell populations were reduced to 0% (Table 1; Figure S5 in Supplementary Material); therefore, the results represent the changes in anti-parasitic potential when specific cell populations are depleted or significantly decreased. The high larvicidal effect observed with the incubation of lung cells and immune serum from the Sm-Cathepsin B + Montanide group was lost upon the depletion of NK cells. As previously mentioned, the necessity of immune serum for the development of high larvicidal activity suggested an ADCC mechanism. Unlike other hematopoietic cells, NK cells do not express inhibitory FcγRs; therefore, possessing only their activating FcγRIIIa, they are free to act as key mediators of ADCC (40). NK cells meditate ADCC by the exocytosis of cytotoxic granules containing perforin and granzyme, or by the release of pro-inflammatory cytokines. The cytotoxic molecules cause direct damage to the target whereas pro-inflammatory cytokines activate nearby immune cells and promote dendritic cell maturation as well as antigen presentation (41). Based on the observations from the *in vitro* schistosomula killing assays, ADCC mediated by NK cells may be the mechanism of protection elicited by Sm-Cathepsin B + Montanide immunizations.

The *in vitro* parasite killing observed with cells taken from the Sm-Cathepsin B + Montanide group was also lost upon the depletion of CD4^+^ T cells. Activated CD4^+^ T cells are an important source of IL-2 which potentiates robust activation of NK cells (42). Activation by IL-2 results in NK cell proliferation, secretion of effector molecules, and enhancement of cytotoxic function (42). NK cell activation *via* CD4^+^ T cell-derived IL-2 may be necessary for schistosomula killing. The importance of CD4^+^ T cell presence for NK cell-mediated mechanisms has been demonstrated for other parasitic infections. NK cell responses targeting *Leishmania major* require IL-2 from primed antigen-specific CD4^+^ T cells (43). Similarly, NK cell targeting of *Plasmodium falciparum* infected red blood cells is dependent on IL-2 from antigen-specific CD4^+^ T cells (44). The data from this present study suggest that protection induced by Sm-Cathepsin B + Montanide immunizations involves the killing of schistosomula *via* ADCC mediated by NK cells that are activated by CD4^+^ T cells.

For the Sm-Cathepsin B + CpG group, the high larvicidal effect was entirely abolished upon the depletion of CD8^+^ T cells, suggesting a main protective effector role for this cell population. As previously mentioned, CpG dinucleotides are known to promote cellular responses such as those including CTLs. CTLs mediate killing of extracellular pathogens by direct recognition, and this function is most often associated with CD8^+^ cells (45). Granulysin has been described as the major effector molecule mediating CTL activity as it inserts into the target membrane and disrupts its permeability (46). CTLs can directly kill *S. mansoni* schistosomula in a contact-dependent manner (47) and can target extracellular *Toxoplasma gondii* in an antigen-specific manner (48). The data from this study suggests that CD8^+^ T cells are the main effectors mediating protection elicited by Sm-Cathepsin B + CpG immunizations. The increased larvicidal activity observed with the Sm-Cathepsin B + CpG group was also significantly decreased upon the depletion of NK cells. However, the parasite killing observed with this experimental group was still higher than the control groups. Therefore, although CD8^+^ T cells could be the main effectors mediating protection, the data suggest that NK cells play an important supportive role in the network of coordinated immune cells. NK cells are important in schistosomiasis progression and protection, and animal models have shown that NK cells activated during schistosome infection negatively regulate granuloma size as well as liver fibrosis (49–51). Furthermore, field studies in schistosomiasis endemic regions have shown that NK cells are linked to schistosomiasis protection in elderly individuals in Brazil (52), while in Sudan, impaired NK cell activity showed a direct relationship with patients’ parasite loads (53).

The importance of NK cells has also been investigated in the context of immunizations against schistosomiasis. It has been demonstrated that protective immune responses elicited by the immunization of mice with lung stage larval antigens + IL-12 depends upon the presence of NK cells (54). In the presented study, larvicidal activity with cells from the Sm-Cathepsin B + CpG group was significantly impaired by the depletion of NK cells. In both cases, the vaccine formulations elicited strong Th1 cellular responses (19, 54). NK cells likely represent the major source of early IFN-γ production. NK cell-derived IFN-γ is believed to regulate IL-12 expression in the context of schistosomiasis as the induction of this cytokine is completely blocked in animals depleted of either IFN-γ or NK cells (49). Since IL-12 is a potent stimulator of CD8^+^ cells, we suggest that early NK-derived IFN-γ triggers IL-12 expression, and together these cytokines prompt CD8^+^ CTL activity targeting schistosomula.

It has been shown that Sm-Cathepsin B has inbuilt adjuvant properties, and that immunizations with antigen alone are capable of significantly reducing parasite burden (18). However, these immunizations elicit low levels of antigen-specific total IgG (<3,500) (18). In the mouse model, the formulation of Sm-Cathepsin B alone stimulates a Th2-biased immune response characterized by increased secretion levels of IL-4, IL-5, and IL-13, but no detectable levels of antigen-specific IgE (18). In the present study, it was demonstrated that incubation of schistosomula with CD45^+^ lung cells from Sm-Cathepsin B immunized animals resulted in parasite killing levels that were significantly higher than those observed for the saline and CpG controls, but still lower than the Sm-Cathepsin B + CpG group. The larvicidal effect was independent of the presence of immune serum. These results suggest that immunizations with unadjuvanted Sm-Cathepsin B stimulate a cellular response capable of targeting the parasite larva. Depletion of CD4^+^ T cells abolished the larvicidal effect observed with the unadjuvanted Sm-Cathepsin B group. This observation suggests that CD4^+^ T cells are important effectors mediating protection elicited by unadjuvanted Sm-Cathepsin B immunizations. In their study, El Ridi and colleagues suggested that immunizations with unadjuvanted Sm-Cathepsin B boost early adaptive immune responses with CD4^+^ T cell help (18). CD4^+^ T cells could potentially be the major source contributing to the observed increase in Th2 cytokines (18). The same research group also suggested that schistosomula will succumb if met by a type 2 cytokine environment (55). One mechanism which they proposed involved the recruitment of eosinophils as well as basophils to the lungs, and their activation by type 2 cytokines (55). The presented study yielded an unexpected result upon the depletion of F4/80^+^ cells. Depleting these cells from the unadjuvanted Sm-Cathepsin B group led to a significant increase in schistosomula killing. This observation may indicate a function for macrophages in limiting effector cell-mediated parasite killing, or it may simply be a result of a significant change in cell population proportions in our *in vitro* assay. Interestingly, the interplay between alternatively activated macrophages and Th2 CD4^+^ T cells has been evaluated in the context of schistosomiasis. The Th2 cytokines IL-4 and IL-13 are known to initiate the alternative activation of macrophages. Arginase 1, a key marker of alternatively activated macrophages, converts l-arginine into l-ornithine and urea. Macrophages expressing this enzyme can successfully compete with Th2 CD4^+^ T cells for arginine; thus, limiting T cell proliferation (56, 57).

In the presented study, it was shown that three different formulations containing the same antigen elicit different protection mechanisms in a mouse model of schistosomiasis. Although the formulations generated similar protection levels, the immune responses they stimulated were different, and this is reflected in the diverse effectors that mediate protection in the immunized animals. In the future, the combination of the different adjuvants, CpG and Montanide ISA 720 VG, will be tested as this could significantly improve a vaccine’s protective potential. Therefore, understanding the underlying mechanisms of vaccine-induced protection will allow for the selection of a formulation that can stimulate the most optimal immune response.

## Ethics Statement

All animal procedures were performed in accordance with Institutional Animal Care and Use Guidelines, and the Animal Use Protocol 7625 was approved by the Animal Care and Use Committee at McGill University.

## Author Contributions

The experiments were designed by MN and AR. JD provided the initial tools needed for protein expression. AR performed all of the experiments. NZ and KV helped with the animal immunizations and cell isolations/depletions. The manuscript was prepared by AR and MN, with revisions by JD.

## Conflict of Interest Statement

The authors declare that the research was conducted in the absence of any commercial or financial relationships that could be construed as a potential conflict of interest.
