# Supplementary material for: Immune Mechanisms Involved in Schistosoma mansoni-Cathepsin B Vaccine Induced Protection in Mice
Source: Front Immunol. 2018 Jul 25;9:1710. doi: 10.3389/fimmu.2018.01710 (PMC6068236; doi:10.3389/fimmu.2018.01710)
Supplement: Supplementary file 1 [file Data_Sheet_1.PDF]

Supplemental figures

A

B

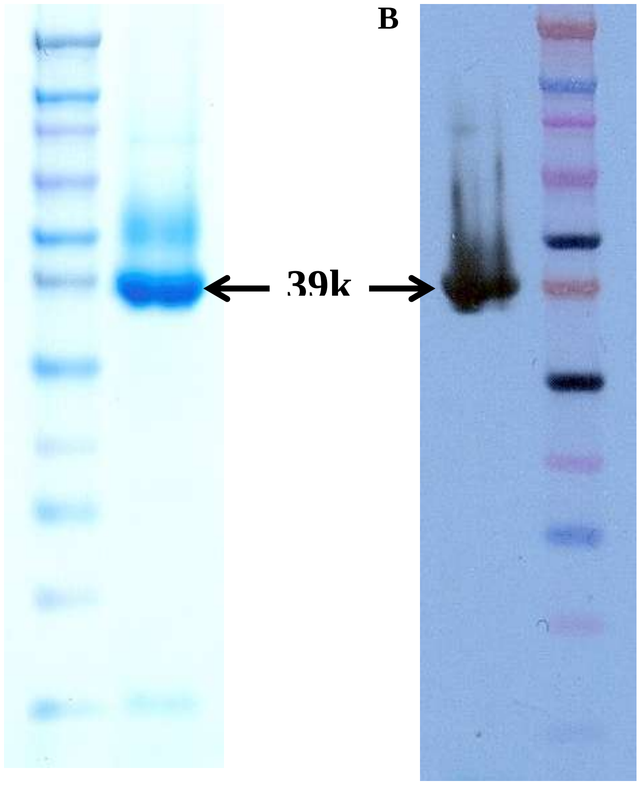

Supplemental Figure 1 Expression of recombinant Sm-Cathepsin B

Recombinant Sm-Cathepsin B was expressed using the PichiaPink™ expression system and purified by Ni-NTA chromatography. Protein expression was analyzed by Coomassie blue staining of polyacrylamide gel **(A)** and western blot **(B)**. The expected band at 39kDa can be observed.

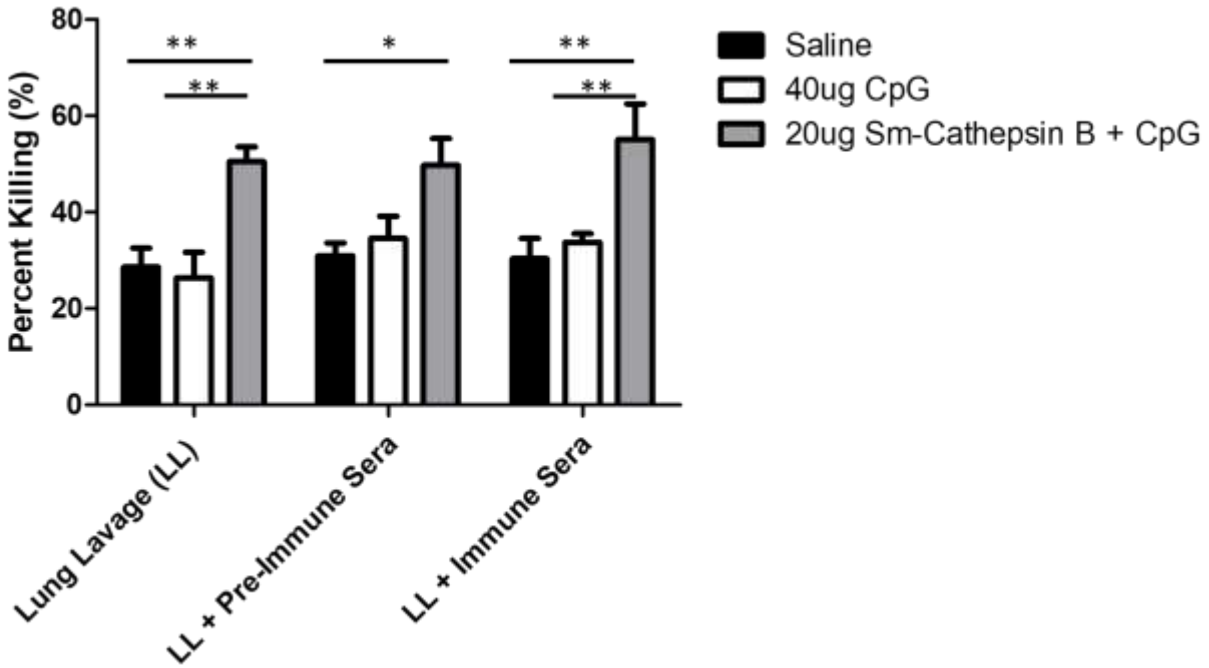

Supplemental Figure 2 Schistosomulae death caused by lung lavage cells taken from mice vaccinated with Sm-Cathepsin B + CpG

Schistosomula were incubated for 24 hours at 37 ºC, 5% CO_2 with serum and cells taken from mice immunized with saline alone, CpG alone, or Sm-Cathepsin B + CpG. Incubations with lung lavage cells were compared to those with whole lung cells. Significant parasite killing is observed with cells taken from the Sm-Cathepsin B + CpG immunized mice, compared to the saline and adjuvant control group mice, in the presence or absence of serum. n = 5. Statistical analysis was performed by 2-way analysis of variance and Bonferroni post test. *: $p\leq 0.05$, **: $p\leq 0.01$.

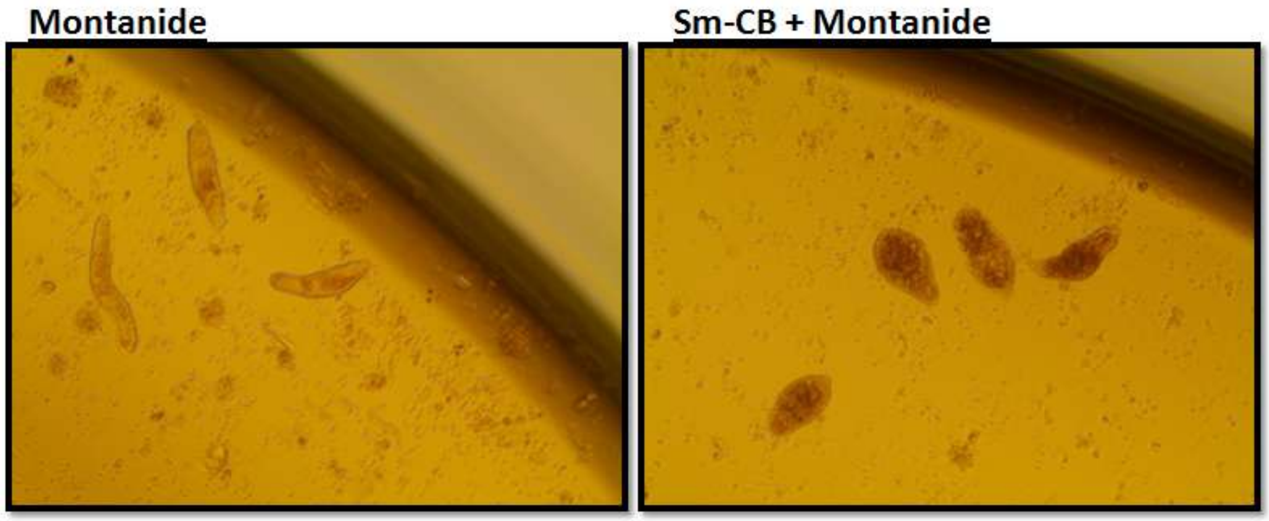

Supplemental Figure 3 Microscopic examination of schistosomula death caused by serum and lung cells taken from mice vaccinated with Sm-Cathepsin B + Montanide.

Schistosomula were incubated for 24 hours at 37 ºC, 5% CO_2 with cells and serum from mice immunized either with Montanide ISA 720 VG or Sm-Cathepsin B + Montanide ISA 720 VG. Parasite viability was determined by microscopic examination of motility, granularity, and shape integrity.

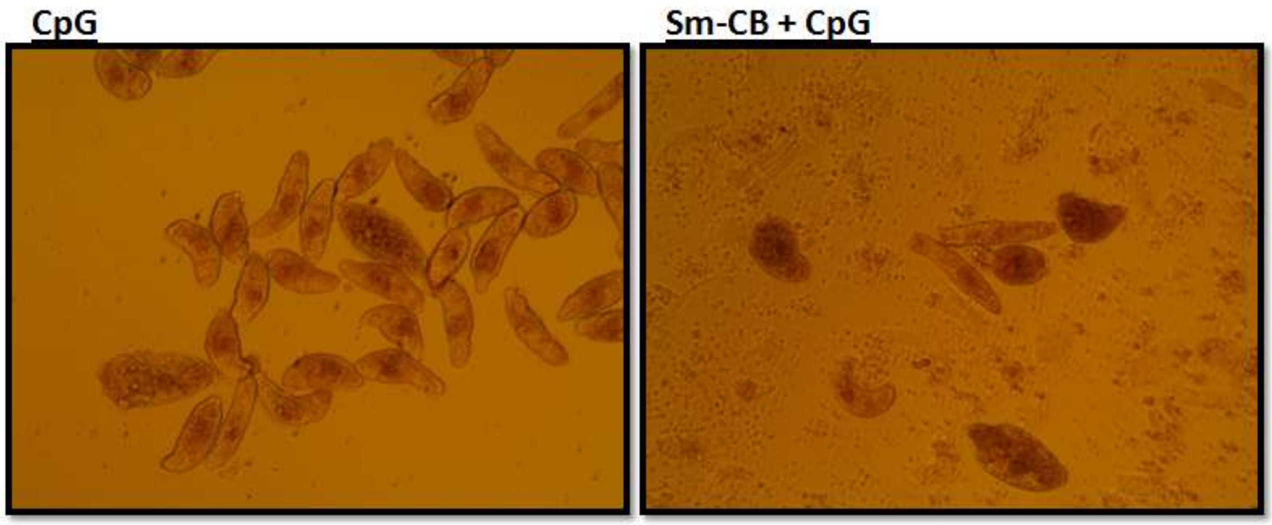

Supplemental Figure 4 Microscopic examination of schistosomula death caused by lung cells taken from mice vaccinated with Sm-Cathepsin B + CpG.

Schistosomula were incubated for 24 hours at 37 ºC, 5% CO_2 with cells and serum from mice immunized either with CpG or Sm-Cathepsin B + CpG. Parasite viability was determined by microscopic examination of motility, granularity, and shape integrity.

A

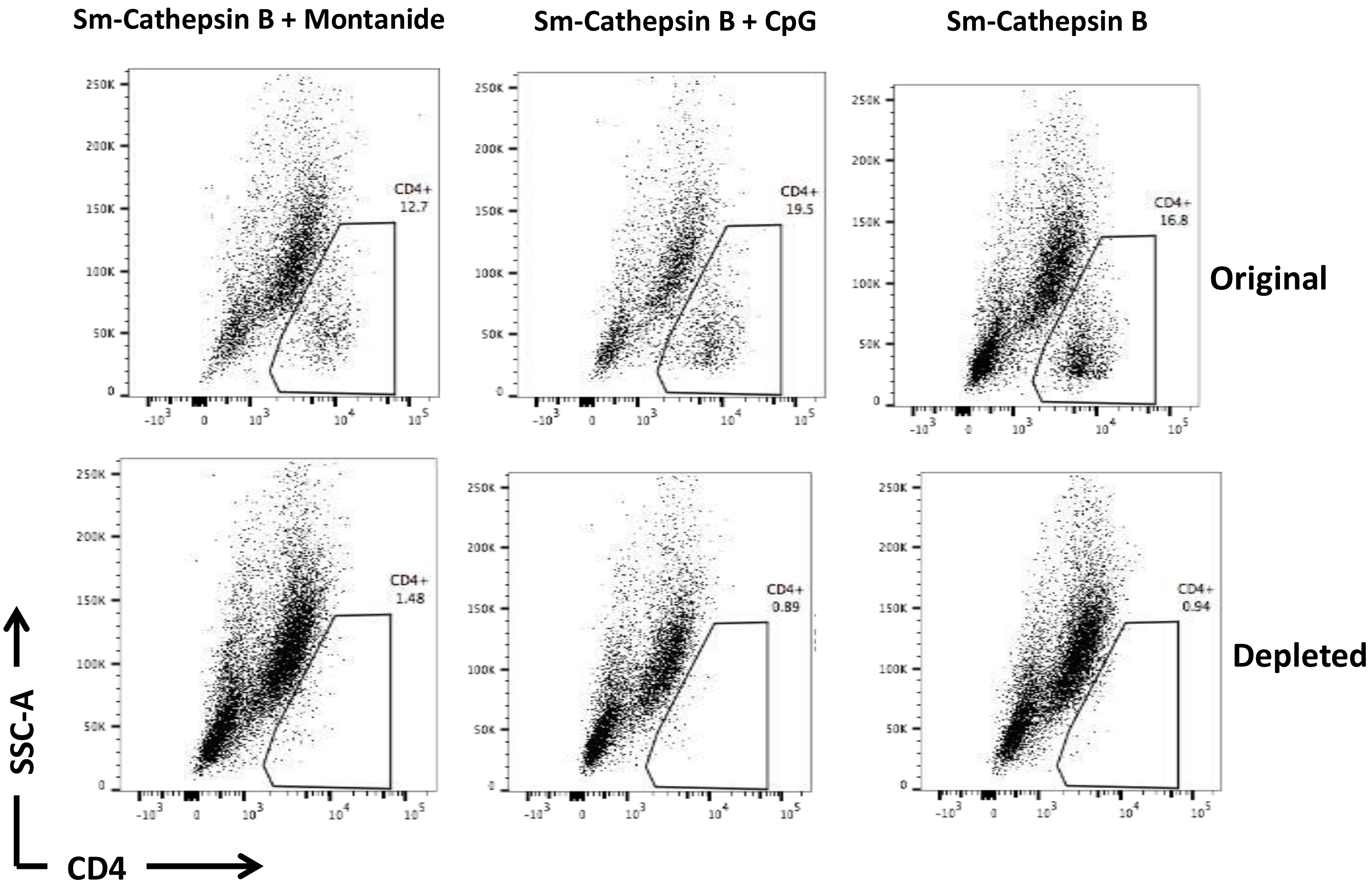
Sm-Cathepsin B + Montanide
Sm-Cathepsin B + CpG
Sm-Cathepsin B
CD4+ 12.7
CD4+ 19.5
CD4+ 16.8
Original
CD4+ 1.48
CD4+ 0.89
CD4+ 0.94
Depleted
SSC-A
CD4

B

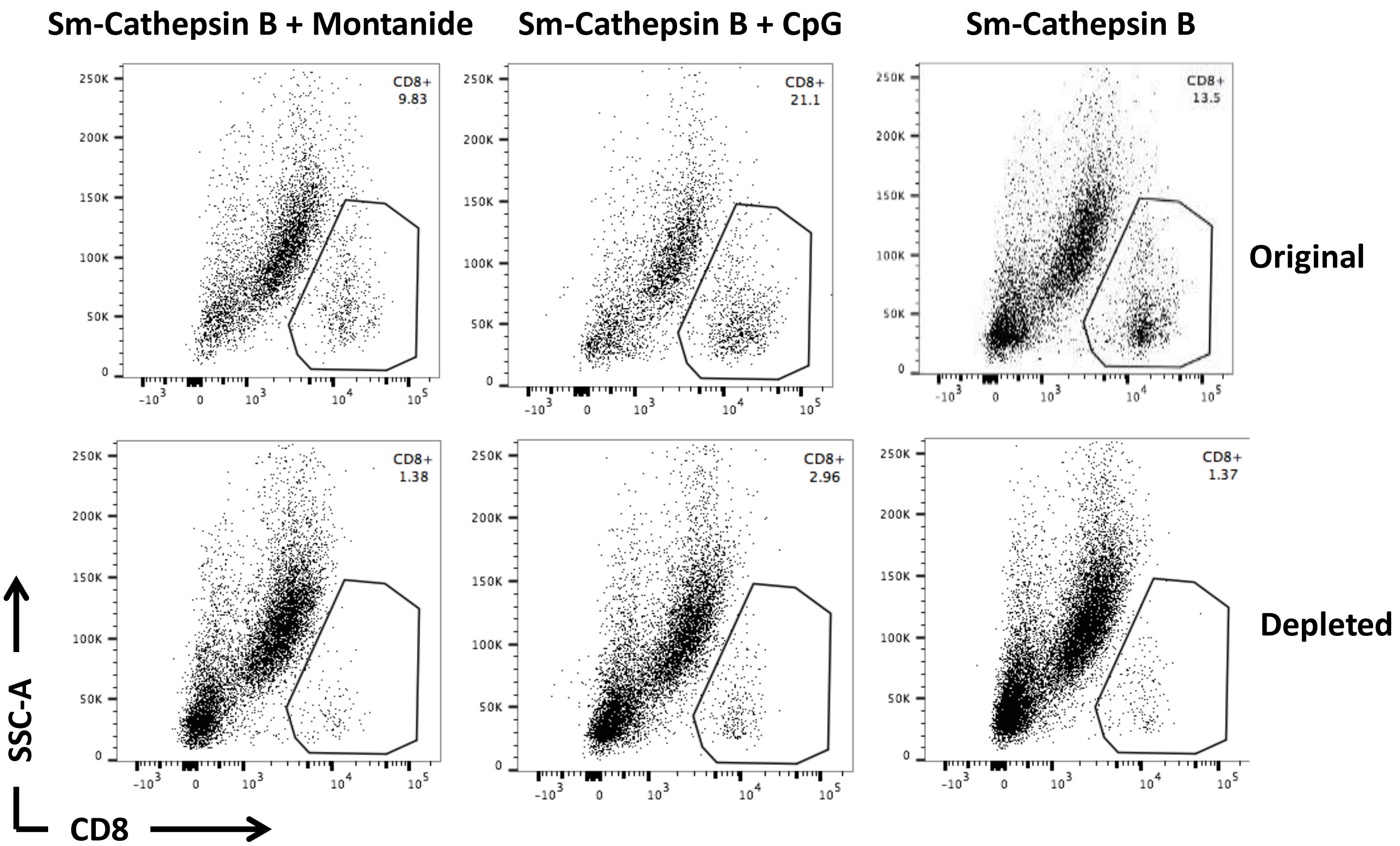
Sm-Cathepsin B + Montanide
Sm-Cathepsin B + CpG
Sm-Cathepsin B
CD8+
9.83
CD8+
21.1
CD8+
13.5
Original
CD8+
1.38
CD8+
2.96
CD8+
1.37
Depleted
SSC-A
CD8

C

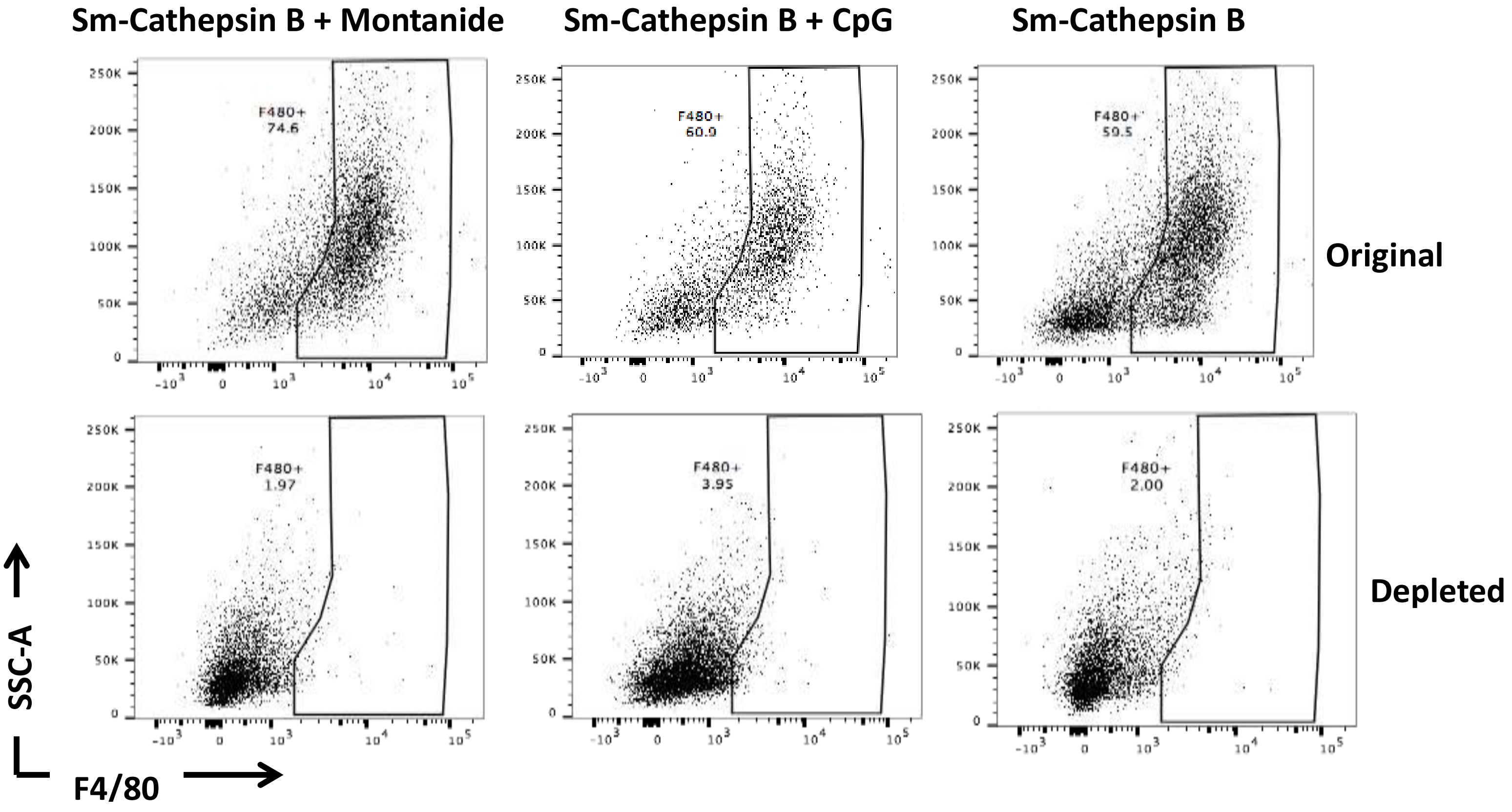
Sm-Cathepsin B + Montanide
Sm-Cathepsin B + CpG
Sm-Cathepsin B
F480+ 74.6
F480+ 60.9
F480+ 59.5
Original
F480+ 1.97
F480+ 3.95
F480+ 2.00
Depleted
SSC-A
F4/80

D

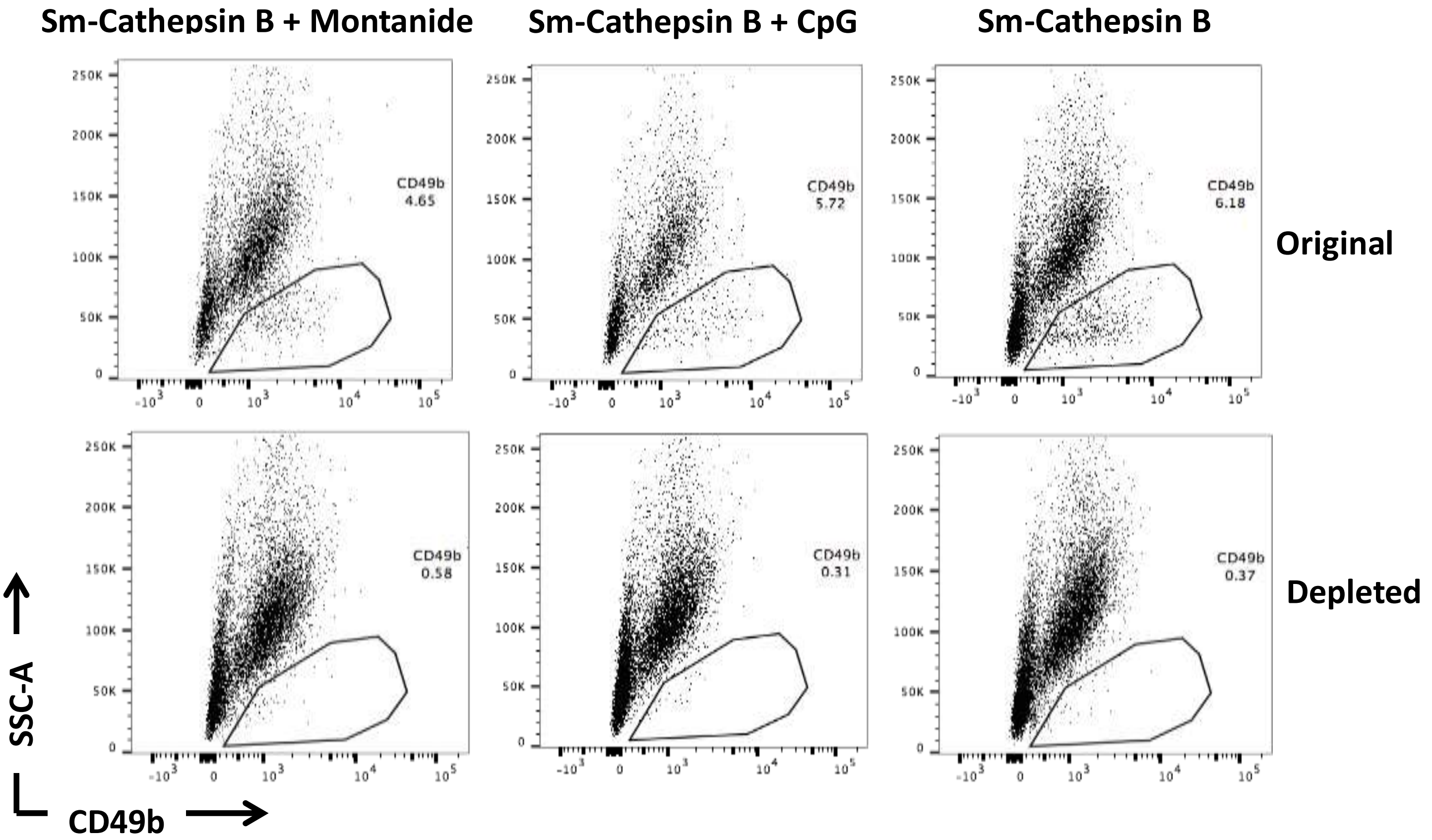

Supplemental Figure 5 Cell population depletion confirmations by flow cytometry.

Lung cell suspensions from the different immunization groups underwent specific depletions followed by $CD45^+$ purification. The depletions were confirmed by flow cytometry. The gates were set to select live $CD45^+$ cells. The top panels represent the original undepleted CD45 purified lung cells taken from Sm-Cathepsin B + Montanide, Sm-Cathepsn B + CpG, and Sm-Cathepsin B immunized mice. The bottom panels represent the lung cell samples from the same immunization groups after the depletions. The depletions were performed and confirmed for **(A)** $CD4^+$, **(B)** $CD8^+$, **(C)** $F4/80^+$, and **(D)** $CD49b^+$ cell populations.
